# Prevalence of anemia and association with mortality in community-dwelling elderly in Thailand

**DOI:** 10.1038/s41598-022-10990-7

**Published:** 2022-04-30

**Authors:** E. Karoopongse, V. Srinonprasert, C. Chalermsri, W. Aekplakorn

**Affiliations:** 1 Department of Medicine, Siriraj Hospital, Mahidol University, Bangkok, 10700 Thailand; 2 Department of Preventive and Social Medicine, Siriraj Hospital, Mahidol University, Bangkok, 10700 Thailand; 3 Department of Community Medicine, Ramathobodi Hospital, Mahidol University, Bangkok, 10400 Thailand

**Keywords:** Geriatrics, Quality of life

## Abstract

Anemia is one of the most common health problems in the elderly in low and middle income countries. Evidence from studies in high income countries suggests that the presence of anemia may predict mortality. We aimed to estimate the prevalence of anemia and the determine the relationship of hemoglobin, mean corpuscular volume (MCV) and mortality in community dwelling Thai elderly. Data from subjects aged ≥ 60 years from the Fourth Thai National Health Examination Survey were analyzed. Comorbidity and hematologic indexes including MCV were obtained. The Cox proportional hazard model was applied to explore associations with mortality. Data from 8,935 subjects were obtained. The mean age of participants was 69.2 years (SD 6.8). 3446 (38.2%) of subjects had anemia; 1931(56%) of these were classified as mild and normocytic. With a total 51,268 person-year of follow up, 753 participants with anemia died, and the cumulative all-cause mortality was 38.5 per 1,000 person-years. The presence of anemia was associated with an increased risk of mortality with HR of 1.66 (95% CI = 1.50–1.84 , p < 0.001). Among subjects with low MCV, hemoglobin level < 10 g/dl in men and < 9 g/dl in women significantly increased the risk of mortality (HR of 2.71, 95% CI = 1.88–3.91 and HR of 3.14, 95%CI = 2.11–4.67, respectively) Persons with anemia and normal MCV, the association with mortality was evident at hemoglobin levels below 11 g/dl for both males and females. (HR of 1.98, 95% CI = 1.67–2.35). Anemia is a moderate to severe public health significant in the population for community dwelling elderly in Thailand. At the same level of Hemoglobin, low MCV population seem to have lower mortality rate than normal MCV. Systematic screening for anemia should be implemented to identify patients at increased risk of mortality. The future research should be focus on causes of anemia and factors contributing to increased mortality in normal to high MCV would be of interest. If this could lead to identifying modifiable causes, it would be beneficial for improving mortality risk among older people.

## Background/introduction

Anemia is a global public health problem^1^. The World Health Organization defines anemia as a hemoglobin level less than 13 g/dl in males and 12 gm/dl in females^2^. A systematic review in mostly higher income countries reported the prevalence of anemia among community dwelling seniors to be around 12%^3^. However the definition of anemia in healthy seniors remains controversial^4–6^; some experts have argue that the definition should be linked to clinical consequences. Multiple studies have reported an association between low hemoglobin and impaired function, increased hospitalization, decreased quality of life, and higher mortality in seniors^5,7–9^. However, most studies use different hemoglobin levels associated with increased risks of negative consequences. A precise cutoff number of hemoglobin in older people would be valuable for practicing physicians.

Around one-third of older adults with anemia are ultimately classified as unexplained anemia of the elderly (UAE)^10^ and require no specific treatment. Red blood cell volume (mean corpuscular volume, MCV) is a commonly available parameter that can be used to investigate the cause of anemia in older people^11,12^. An index of hemoglobin levels stratified by MCV could be useful to guide treatment decisions. Few studies have reported on the prevalence of anemia in community dwelling seniors in Thailand. Moreover, studies of the association between hemoglobin levels and mortality among older people have been limited. The optimal level of hemoglobin for people with low MCV that is associated with increased mortality is of particular interest.

## Materials and method

The Thai National Health Examination Survey (NHES-IV) is a nationally representative survey using a multistage, stratified sampling of the Thai population. The detail of sampling methods including mortality data has been described elsewhere^13,14^.This study included data from 8,935 participants aged 60 years and older who had their blood tested to assess hemoglobin levels. The baseline assessment was conducted from August 2008 to March 2009. Participants were asked to fast 12 h overnight before the venous blood was obtained the next morning. A complete blood count was performed using whole venous blood collected in 3 mL EDTA (Ethylene Diamine Tetraacetic Acid) tubes and processed with an automated system. Hemoglobin level and all indexes including MCV were measured with the Sysmex XE-5000 hematology analyzer using the manufacturer’s reagents and methods. The details of the sampling method of this cohort including data collection and mortality data were prescribed elsewhere.^15,16^.

### Data collection and measurement

All information was obtained through semi-structured interviews using standardized questions. Demographic and socioeconomic data such as age, gender, smoking, area of residence, geographic region were collected through interviewing participants. Trained research staff interviewed participants for basic activities of daily living (BADLs), physical activities, food and alcohol consumption frequency and medical comorbidities such as stroke, cardiovascular diseases and COPD. The weekly frequency of physical activities was assessed using the Global Physical Activity Questionnaire version 2. Bodyweight and height were measured by standardized procedures and body mass index (BMI) was calculated.

Hypertension was defined as systolic blood pressure (SBP) ≥ 140 mmHg, diastolic blood pressure (DBP) ≥ 90 mmHg or self-reported use of blood pressure-lowering medications. Diabetes was defined as a self-reported diagnosis of diabetes, use of antidiabetic agents, or fasting plasma glucose ≥ 7.0 mmol/L from venous blood drawn during the assessment. Chronic kidney disease (CKD) was defined as individuals who self-reported receiving dialysis or who had a glomerular filtration rate < 60 mL/min calculated using the Chronic Kidney Disease Epidemiology Collaboration (CKD-EPI) Eq. ^17^.

BMI was classified using the Asia–Pacific cut-off values: BMI < 18.5 kg/m2 as underweight and ≥ 25.0 kg/m2 as obese. Having impaired basic-ADL was defined as any response indicating that the respondent required assistance in performing any basic-ADL. The level of physical activity was classified into low, intermediate and high using tertile cutoffs. Red meat consumption was selected to be representative of food consumption and defined as low and high levels of consumption.

Anemia was classified based on mean corpuscular volume (MCV). Microcytic or low MCV anemia is defined by MCV on the complete blood count (CBC) under 80 famtoliter (fL) while normal MCV anemia (normocytic anemia) defined by MCV ranged between 80–100 fL. Macrocytic anemia is defined by MCV larger than 100 fL.^18^ The severity of anemia was classified based on WHO classification which was divided into severe anemia if Hb less than 8 g/dl, moderate anemia if Hb between 8–11 g/dl and mild anemia if Hb between 11–12 g/dl in female and 11–13 g/dl in male^19^.

Mortality data was drawn from the Thai vital registration system whereby all death is mandated by law to be registered to this registration. Mortality data was retrieved until May 2016. The death registration has been ascertained to be a reliable source of death statistics in the country^20^. This study was approved by the ethics committee of the Faculty of Medicine, Ramathibodi Hospital and the Faculty of Medicine, Siriraj Hospital, Mahidol University. Written informed consent was obtained from all subjects during the initial assessment. The SIRB is grounded in foundational ethical principles embodied in the Declaration of Helsinki of 1964 and its subsequent revisions and the Belmont Report.

### Statistical methods

Descriptive statistics were used to compare baseline characteristics of participants. Categorical data are reported as frequency and proportion, and continuous data are given as mean ± standard deviation or median with minimum and maximum depending on the distribution of data. Analysis of variance (ANOVA) test was applied for continuous variables, while chi-square test was used for categorical variables. The distribution of hemoglobin level and MCV were calculated and demonstrated to identify the level of hemoglobin at the 5 percentile for each gender. A *p*-value less than 0.05 was considered statistically significant.. The proportional hazard assumption was tested and the result was not violated. Cox regressions were conducted to determine the unadjusted hazard ratios (HRs) for mortality. Potential confounders of all-cause mortality among participants with anemia included in the analysis were age, gender, current smoking status, hypertension, diabetes, living in urban areas, low BMI, basic activity of daily living, physical activity, red meat and alcohol consumption. Variables that had a p < 0.1 in univariate analyses were then included in a Cox proportional hazards model. Statistical analyses were performed using STATA 15.0 (StataCorp LP, College Station, TX, USA).

## Result

The mean age of the 8,935 subjects was 69.5 years (SD = 6.8). The overall prevalence of anemia was 38.2% (women 42.4%; men 35.8%). The prevalence of anemia was 32.3% in the 60–69 year age group; 45.0% in the 70–79 year age group, and 56.2% in participants > 80 years. The majority (84.4%) had mild anemia, while 13.3% had moderate and 0.02% had severe anemia. 6,621 subjects (74.1%) had normocytic anemia, 2198 subjects (24.6%) had microcytic anemia and 134 subjects (1.5%) had macrocytic anemia. In people with severe anemia, microcytic anemia is the most common type. Normocytic anemia is the most common type of anemia in older people with moderate anemia. Anemic participants were more likely to have impaired activity of daily living, low BMI and reduced eGFR. (Table 1).

**Table 1:** Basline characteristic of included population.

Baseline characteristic	Total population	Non-anemic population	Anemic population	
			MCV < 80 fL (Microcytic anemia)	MCV 80–100 fL (Normocytic anemia)
Number (n)	8869	5438	1378	2053
Age, year Mean(SD)	69.4 (7.0)	68.4 (6.6)	70.3 (7.3)	71.6 (7.2)
Hypertension, n(%)	2963 (33.4)	1796 (33.1)	446 (32.4)	721 (35.1)
DM, n(%)	1426 (16.4)	826 (15.5)	225 (16.7)	375 (18.7)
CVA, n(%)	321 (3.6)	189 (3.5)	48 (3.5)	84 (4.1)
COPD, n(%)	205 (2.3)	118 (2.1)	33 (2.4)	54 (2.6)
CKD, n(%)	2622 (29.6)	1245 (22.9)	490 (35.6)	887 (43.2)
**BMI,n(%)**				
Low BMI, n(%)	1163 (13.3)	589 (10.9)	217 (16.0)	357 (17.7)
Normal BMI, n(%)	4817 (55.1)	2865 (53.2)	802 (59.3)	1150 (57.3)
Obesity, n(%)	2764 (31.6)	1931 (35.8)	333 (24.6)	500 (24.9)
Urban, n(%)	4745 (53.5)	3034 (55.7)	655 (47.5)	1056 (51.4)
High meat intake, n(%)	2512 (28.3)	1619 (29.7)	402 (29.1)	491 (23.9)
Current alcohol consumption, n(%)	2498 (28.2)	1664 (30.6)	344 (25.0)	490 (23.9)
Impaired BADLs, n(%)	4104 (47.0)	2318 (43.3)	687 (50.6)	1099 (54.5)
**Physical activity, n(%)**				
Low physical activity, n(%)	2658 (30.4)	1507 (28.0)	428 (31.4)	723 (35.6)
Moderate physical activity, n(%)	2845 (32.5)	1747 (32.5)	433 (31.8)	665 (32.8)
High physical activity, n(%)	3250 (37.1)	2112 (39.4)	500 (36.7)	640 (31.5)
Death, n, (per 1000 person-year)	1479 (29.1)	741 (23.3)	264 (33.9)	474 (41.7)

Additional analysis was carried out to explore the distribution of hemoglobin level and MCV among healthy older people. This was investigated among older people without chronic diseases known to have an effect on hemoglobin level and results are shown in Table 2.

**Table 2:** Distribution of hemoglobin and MCV in community dwelling older persons without known comorbid diseases.

Hemoglobin level (g/dl)	Min,max	5 centile	25 centile	50 centile	75 centile	95 centile	Mean	SD
**Male**								
Normal MCV	8.6, 19.1	11.7	13.0	13.9	14.8	16.0	13.9	1.36
Low MCV	5.0, 16.7	9.7	11.7	12.7	13.6	14.9	12.5	1.65
**Female**								
Normal MCV	5.8, 17.2	10.8	11.9	12.7	13.4	14.4	12.6	1.12
Low MCV	7.0, 17.6	9.4	10.8	11.6	12.4	13.6	11.6	1.33
**MCV (fl)**								
Male	50.0, 110.2	67.0	80.7	87.2	91.9	97.6	85.5	9.17
Female	52.2, 112.4	66.3	79.9	85.9	90.4	95.5	84.3	8.64

Overall, ranges of hemoglobin level in apparently healthy older persons in community were lower among female compared to male.

According to WHO report^2^, levels of hemoglobin for defining anemia were made to expect the proportion of population having hemoglobin below those levels at 5%.

Focusing on normal MCV group, levels of hemoglobin for people at the lowest 5 percentile of the study population were 11.7 g/dl for male and 10.8 g/dl for female. For participants with low MCV, the hemoglobin at level of 5 percentile should be 9.7 g/dl for male and 9.4 g/dl for female which were lower than definitions of anemia suggested by WHO^2^.

### Anemia, hemoglobin level and survival among male and female

**Figure 1:**
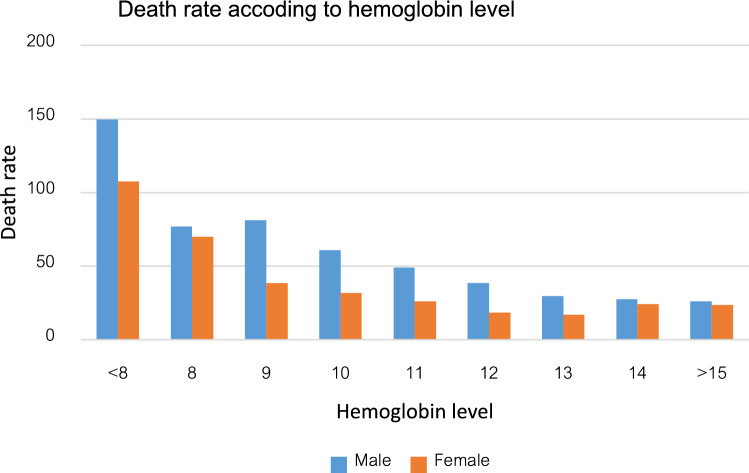
Death rate increases with lower level of hemoglobin in both gender.

During the 51,268 person-years of follow-up, 753 participants with anemia died, and the cumulative all-cause mortality was 38.5 per 1,000 person-years. Lower hemoglobin levels were associated with increased mortality for both male and female (Fig. 1). The death rate increased as hemoglobin levels decreased and was more pronounced in males compared to females. The presence of anemia was associated with increased mortality with HR of 1.66 (95% CI = 1.50–1.84 , p < 0.001) for the entire cohort.

Figure 1 The death rate increased constantly with hemoglobin level below definition of anemia according to WHO criteria.

In participants of both genders with normal MCV, the level of hemoglobin associated with increased mortality was higher compared to participants with low MCV. For anemic subjects with normal MCV, a hemoglobin level of 10–11 g/dl was statistically associated with increased mortality with a HR of 1.72 (95% CI = 1.12–2.64) in men and 1.41 (95% CI = 1.01–1.98) in women. For anemic subjects with low MCV, a hemoglobin level of 9–10 g/dl had a HR of 2.37 (95% CI = 1.20–4.68) in men while a hemoglobin of 8–9 g/dl had a HR of 2.01 (95% CI = 1.06–3.81) in women. The association between hemoglobin and mortality became stronger with lower levels of hemoglobin for both normal and low MCV as shown in Table 3.

**Table 3:** Level of hemoglobin associated with mortality, stratified by sex.

Hemoglobin level (g/dl)	Hazard ratio for men*	Hazard ratio for women*		
	Normal MCV (≥ 80 )	Low MCV (< 80)	Normal MCV (≥ 80 )	Low MCV (< 80)
< 8	3.76 (0.52–27.08)	4.10 (1.87–8.99)**	7.27 (2.92–18.09)**	1.48 (0.66–3.34)
8–9	2.32 (0.94–5.72)	2.02 (0.87–4.69)	2.14 (1.10–4.18)**	2.01 (1.06–3.81)**
9–10	1.67 (0.87–3.19)	2.37 (1.20–4.68)**	1.65 (0.94–2.92)	1.07 (0.59–1.94)
10–11	1.72 (1.12–2.64)**	1.56 (0.87–2.77)	1.41 (1.01–1.98)**	0.81 (0.47–1.39)
11–12	1.32 (0.98–1.78)	1.58 (0.98- 2.55)	1.17 (0.88–1.54)	1.02 (0.64–1.63)
12–13	1.20 (0.95–1.53)	1.24 (0.76–2.04)	**1.00**	**1.00**
13–14	**1.00**	**1.00**	0.98 (0.72–1.34)	1.36 (0.71–2.57)
14–15	1.01 (0.79–1.28)	0.91 (0.48–1.73)	1.40 (0.94–2.09)	2.21 (0.91–5.35)
≥ 15	1.09 (0.83–1.42)	0.92 (0.35–2.41)	1.38 (0.69–2.73)	NA

*Analysis was adjusted for age, diabetes, CKD, BMI, urban area, impaired BADL, Physical activity, alcohol and red meat consumption.

**p-value < 0.05.

**Figure 2:**
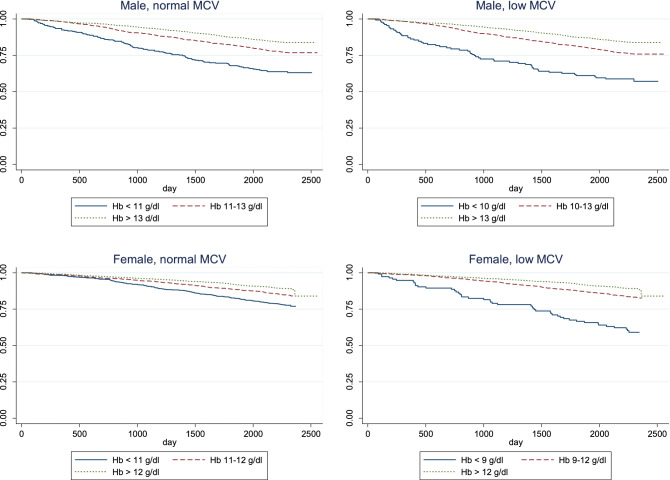
Survival curve according to MCV category for both genders.

The model generated different survival curves in men and women when we stratified groups by MCV utilizing different hemoglobin level cutoffs (Fig. 2). In participants with low MCV, a hemoglobin level between 9–10 g/dl in men and 8–9 g/dl in women were applied as the reference group and demonstrated different mortality risk according to groups. For participants with normal MCV, we used a hemoglobin level of 10–11 g/dl as the reference group, differentiation of survival curves were evidencely more separated among male compared to female.

## Discussion

This is the first nation-wide population-based study of anemia among older people in Thailand demonstrating a very high prevalence of anemia. The prevalence of anemia is substantialy higher than previous studies conducted in high income countries^3,21,22^ but similar to other middle income contries such as China^23^ and Malaysia^24^. Nevertheless, the present study appears to be the first community-base study in the context of low-to-middle income country with long term follow-up to demonstrate the association with increased mortality in anemic older persons. This emphasizes the important of problem that it should be raised as a public health issue requiring urgent attention to seek for appropriate dealing strategies.

We identified different cutoff hemoglobin levels that were associated with increased mortality. Mildly reduced hemoglobin (< 11 g/dl for both genders) was associated with increased mortality in participants with normal MCV, while an Hb < 10 g/dl in men and < 9 g/dl in women was associated with increased mortality in participants with low MCV.

Moreover, when taking survival curves from Fig. 2 into consideration, it could be proposed that anemia with normal MCV among male might be a more a appropriate target to explore the cause and find appropriate public intervention at the community level.

Despite abundant evidence for the association between anemia and mortality among older people^8,12,22,25^, no causal relationship has been established. Anemia may be a marker for other diseases rather than the cause of the increased mortality. Stratified risk of death among older people using MCV might provide some more insight into the association. Increased mortality in anemic people with low MCV could represent iron deficiency anemia or moderate chronic kidney disease that could lead to premature death^26,27^, especially if left untreated. We observed a significantly increased risk of death in anemic participants with normal MCV, which was more pronounced in men.

A substantial proportion of this group could be diagnosed as unexplained anemia of the elderly (UAE), which receives limited attention in current practice as there are no recommended interventions^10^. Studies from the UAE concluded that there are many contributing factors including low testoterone^10,28^. Although the role of testosterone in older men has been debated, evidence suggests that low testosterone levels are associated with unexplained anemia^26^ and increased mortality in older men^29,30^. There has been no long term study of the effect of low testosterone in anemic normal MCV individuals and mortality. Further study is warranted.

The optimal level of hemoglobin in older persons remains unclear^4–6,10,31^. According to the WHO^2^, it was expected that the cutoff value indicated 5 percentile of the population. Following this suggestion, the cutoff values would be 11.7 g/dl for men with normal MCV; 9.7 g/dl for men with low MCV; 10.8 g/dl for women with normal MCV, and 9.4 g/dl for women with low MCV. (Table 2).

However, considering another angle of the cutoff levels which should be the points determining clinical significant such as increased mortality. In our study, we reveal that elderly male participants with Hb less than 11 g/dl with normal MCV and less than 10 g/dl with low MCV experience higher mortality rate. Mean while, the number should be 11 g/dl for female with normal MCV and 9 g/dl for female with low MCV in older people.

Systematic screening for anemia in the elderly is needed/necessary/important. Most older people with anemia are asymptomatic^4^. Older people also tend to underreport their symptoms, even with moderate to severe anemia^12^. Currently, there are no international guidelines to screen for anemia in older people. A mass screening program for asymptomatic older people should be offered for community dwelling older adults, particularly in low-to-middle income countries with a high prevalence of this potentially treatable condition.

The present study has some strengths and limitations. One strength is the use of nationally representative sample of community dwelling seniors. Moreover, the present study introduced MCV-stratified strategies to refine the different levels of risk among older people in the community. The identified levels of hemoglobin are considerably lower than levels recommended by WHO. Using the cutoff values we propose would focus attention on smaller numbers of higher risk populations.

There are several limitations of the study. First, specific causes of anemia and death in participants were not studied. Second, the level of hemoglobin could have change and the magnitude of risk might be different overtime. We could not ascetrtain subsequent anemic status of participants in the cohort which is a common limitation for most cohort studies. Thirdly, we did not explore specific causes of death in this population. Although, providing the fact that most older persons die as a consequence of several factors^31,33^, immediate cause of death may be less explanatory compared to younger population. Factors affecting all cause mortality for older population remains worthwhile exploring. Furthermore, there could be residual confounding factors in our study despite of adjusting for several potential confounding factors. Nevertheless, discovering that lower level of hemoglobin associated with increased all-cause mortality in different analysis models would help affirming the validity of the association. Providing those limitations, future research to focus on causes of anemia and factors contributing to increased mortality in normal to high MCV would be of interest. If this could lead to identifying modifiable causes, it would be beneficial for improving mortality risk among older people.

## Supplementary Information

Supplementary Information 1.

Supplementary Information 2.
