# Supplementary material for: Prevalence of anemia and association with mortality in community-dwelling elderly in Thailand
Source: Sci Rep. 2022 Apr 30;12:7084. doi: 10.1038/s41598-022-10990-7 (PMC9056501; doi:10.1038/s41598-022-10990-7)
Supplement: Supplementary file 1 — Supplementary Information 1. [file 41598_2022_10990_MOESM1_ESM.docx]

**Table S1 : Baseline characteristic and death rate of included male population**

| Variable for male participants | Hemoglobin level (n=4387) | | | | | | | | | p-value |
| --- | --- | --- | --- | --- | --- | --- | --- | --- | --- | --- |
|  | >15 | 14-15 | 13-14 | 12-13 | 11-12 | 10-11 | 9-10 | 8-9 | <8 |  |
| Number (n) | 656 | 1002 | 1178 | 823 | 434 | 162 | 72 | 36 | 24 |  |
| Age,years,  Mean(SD) | 67.0(6.0) | 68.1 (6.4) | 69.4 (6.6) | 70.7 (7.2) | 71.6 (7.2) | 72.4 (7.4) | 72.6 (7.5) | 72.1 (5.8) | 71.0 (7.2) | <0.001 |
| MCV,fl  Mean(SD) | 89.3(5.9) | 88.1(7.1) | 85.9 (7.9) | 84.0 (9.3) | 80.8 (10.5) | 78.2 (11.8) | 75.1 (13.0) | 75.6 (13.1) | 73.5 (14.2) | <0.001 |
| Low MCV (%) | 41(6.4) | 121(12.3) | 246(21.2) | 241(30.0) | 178(41.6) | 86(55.8) | 43(61.4) | 23 (65.7) | 17 (85.0) | <0.001 |
| Smoking ,n(%) | 508 (77.6) | 786(78.6) | 912 (77.6) | 662 (80.5) | 347 (80.3) | 139(85.8) | 59 (81.9) | 26(72.2) | 18(75.0) | 0.265 |
| Impaired BADLs ,n(%) | 221(34.1) | 342(34.6) | 407 (35.2) | 329 (40.6) | 203 (47.7) | 78 (48.8) | 35 (49.3) | 20 (55.6) | 11 (45.8) | <0.001 |
| Hypertension  ,n(%) | 209(32.0) | 278(27.9) | 371 (31.6) | 235 (28.6) | 121 (28.0) | 48 (29.6) | 23 (31.9) | 10 (27.8) | 8 (33.3) | 0.570 |
| DM ,n(%) | 103(16.1) | 127(13.0) | 172 (14.9) | 112 (14.0) | 69 (16.3) | 27 (17.2) | 10 (14.5) | 6 (16.7) | 5 (20.8) | 0.623 |
| CVA,n(%) | 36(5.5) | 42(4.2) | 41 (3.5) | 41 (5.0) | 14 (3.2) | 7 (4.3) | 1 (1.4) | 1 (2.8) | 0 (0.0) | 0.328 |
| COPD,n(%) | 18(2.8) | 36(3.6) | 36 (3.1) | 37 (4.5) | 16 (3.7) | 10 (6.2) | 3 (4.2) | 1 (2.8) | 1 (4.2) | 0.481 |
| CKD ,n(%) | 152(23.2) | 207 (20.7) | 309(26.2) | 256 (31.1) | 179 (41.2) | 68 (41.0) | 47 (65.3) | 18 (50.0) | 12 (50.0) | <0.001 |
| Low BMI ,n(%) | 67(10.3) | 125(12.6) | 158(13.5) | 142(17.5) | 84(19.7) | 41(25.6) | 16(22.5) | 5(13.9) | 7(30.4) | <0.001 |
| Urban ,n(%) | 374(57.0) | 547(54.6) | 612 (52.0) | 429 (52.1) | 210 (48.4) | 73 (45.1) | 31 (43.1) | 23 (63.9) | 10 (41.7) | 0.013 |
| Death, n, (per 1000 person-year) | 99(25.9) | 159 (27.4) | 201 (29.7) | 177 (38.4 ) | 116 (49.1) | 52( 60.8) | 28 (81.1) | 13(76.9 ) | 14 (149.5) | <0.001 |
