# Supplementary material for: Prevalence of anemia and association with mortality in community-dwelling elderly in Thailand
Source: Sci Rep. 2022 Apr 30;12:7084. doi: 10.1038/s41598-022-10990-7 (PMC9056501; doi:10.1038/s41598-022-10990-7)
Supplement: Supplementary file 2 — Supplementary Information 2. [file 41598_2022_10990_MOESM2_ESM.docx]

**Table S2 : Baseline characteristic and death rate of included female population**

| Variable for female participants | Hemoglobin level (n=4548 ) | | | | | | | | | p-value |
| --- | --- | --- | --- | --- | --- | --- | --- | --- | --- | --- |
|  | >15 | 14-15 | 13-14 | 12-13 | 11-12 | 10-11 | 9-10 | 8-9 | <8 |  |
| Number (n) | 73 | 293 | 924 | 1363 | 1091 | 504 | 186 | 77 | 37 |  |
| Age,years,  Mean(SD) | 68.3(6.8) | 68.1(6.8) | 68.0(6.6) | 69.0 (6.9) | 70.3 (7.1) | 71.9 (7.7) | 71.7 (7.4) | 72.2 (7.6) | 74.5 (6.9) | <0.001 |
| MCV,fl  Mean(SD) | 88.2(7.8) | 88.4(5.6) | 87.4(6.1) | 85.5 (7.4) | 82.7 (8.7) | 80.1 (10.2) | 75.7 (10.9) | 75.2 (13.2) | 74.8 (13.7) | <0.001 |
| Low MCV (%) | 8(11.1) | 23(8.0) | 105(11.4) | 270 (19.9) | 361 (33.2) | 217 (44.2) | 122 (67.0) | 50 (67.6) | 26(76.5) | <0.001 |
| Smoking ,n(%) | 24(32.9) | 69(23.6) | 221(24.0) | 318 (23.3) | 270 (24.8) | 154(30.6) | 60(32.3) | 22 (28.6) | 17(46.0) | 0.001 |
| Impaired BADLs ,n(%) | 36(49.3) | 136(47.6) | 465(51.2) | 739(55.0) | 614 (57.1) | 298 (60.3) | 117 (64.6) | 57 (74.0) | 31 (83.8) | <0.001 |
| Hypertension  ,n(%) | 26(35.6) | 117(39.9) | 326(35.3) | 492 (36.2) | 416 (38.2) | 188 (37.4) | 72 (38.7) | 32 (41.6) | 14 (38.9) | 0.828 |
| DM ,n(%) | 13(17.8) | 58(20.4) | 148(16.3) | 219 (16.3) | 203 (19.0) | 107 (21.7) | 41 (22.2) | 17 (22.7) | 2 (5.4) | 0.024 |
| CVA ,n(%) | 3(4.1) | 9(3.1) | 20(2.2) | 43(3.2) | 38 (3.5) | 13 (2.6) | 5 (2.7) | 5 (6.5) | 4 (10.8) | 0.072 |
| COPD ,n(%) | 1(1.4) | 2(0.7) | 9(1.0) | 17 (1.3) | 8 (0.7) | 4 (0.8) | 8 (4.3) | 1 (1.3) | 1 (2.7) | 0.008 |
| CKD ,n(%) | 22(30.1) | 73(24.9) | 173(18.7) | 333 (24.4) | 385 (35.3) | 231 (45.8) | 105 (56.5) | 50 (64.9) | 28 (75.7) | <0.001 |
| Low BMI ,n(%) | 7(9.7) | 27(9.4) | 78(8.5) | 133 (9.9) | 143 (13.4) | 80 (16.4) | 37 (21.0) | 14 (18.4) | 13 (38.2) | <0.001 |
| Urban ,n(%) | 42(57.5) | 181(61.8) | 517(56.0) | 785 (57.6) | 576 (52.8) | 241 (47.8) | 81 (43.6) | 31 (40.3) | 20 (54.1) | <0.001 |
| Death, n, (per 1000 person-year) | 10(23.6) | 42(24.2) | 92 (16.8) | 150 (18.6) | 166 (26.1) | 92 (31.8) | 40(38.3) | 28 (70.0) | 18 (107.5) | <0.001 |
